# Toxic epidermal necrolysis induced by cisplatin for cervical squamous cell carcinoma

**DOI:** 10.1093/skinhd/vzag059

**Published:** 2026-05-04

**Authors:** Roxani Kapranou, Liberis Louros, Stefanos Tsallas, Irene Gamatsi, Athanasios Karonidis, Maria Kostaki

**Affiliations:** General Hospital of Athens “Georgios Gennimatas”, Plastic Surgery, Microsurgery, Burns and Melanoma Referral Center, Athens, Greece; General Hospital of Athens “Georgios Gennimatas”, Plastic Surgery, Microsurgery, Burns and Melanoma Referral Center, Athens, Greece; General Hospital of Athens “Georgios Gennimatas”, Plastic Surgery, Microsurgery, Burns and Melanoma Referral Center, Athens, Greece; General Hospital of Athens “Georgios Gennimatas”, Plastic Surgery, Microsurgery, Burns and Melanoma Referral Center, Athens, Greece; General Hospital of Athens “Georgios Gennimatas”, Plastic Surgery, Microsurgery, Burns and Melanoma Referral Center, Athens, Greece; General Hospital of Athens “Georgios Gennimatas”, Plastic Surgery, Microsurgery, Burns and Melanoma Referral Center, Athens, Greece

## Abstract

Stevens-Johnson syndrome/toxic epidermal necrolysis (SJS/TEN) are rare but potentially life-threatening adverse drug reactions, characterized by widespread necrosis and detachment of the epidermis. Only a few cases in the literature have linked this severe drug reaction to chemotherapy agents. Herein, we report a case of TEN that developed following chemotherapy with cisplatin for cervical squamous cell carcinoma.

Dear Editor, Stevens–Johnson syndrome/toxic epidermal necrolysis (SJS/TEN) are rare but potentially life-threatening adverse drug reactions, characterized by widespread epidermal necrosis and subsequent epidermal detachment.^1^ These conditions are considered part of a disease continuum: SJS involves <10% epidermal detachment of the body surface area (BSA), SJS/TEN overlap 10–30% of BSA epidermal detachment and TEN >30% of BSA epidermal detachment.^2^ Commonly implicated triggers include antibiotics (such as sulfonamides and penicillins) and antiepileptics (carbamazepine, phenytoin and lamotrigine).^2^ Immunotherapy agents such as immune checkpoint inhibitors have also been associated with the development of SJS/TEN-like reactions.^1^ Infections with *Mycoplasma pneumoniae*, for example, have rarely been implicated in SJS/TEN, and in some cases no identifiable trigger is found.^1^ Herein, we report a case of TEN that developed following chemotherapy with cisplatin for cervical squamous cell carcinoma (SCC).

A 47-year-old woman underwent concurrent chemoradiation for newly diagnosed locally advanced cervical SCC [Fédération International de Gynécologie et Obstétrique (FIGO) staging IIA; T2aN0M0]. Two weeks after the first radiotherapy, a weekly cisplatin treatment was initiated (30 mg m^–2^). Six days after the second cisplatin cycle she developed fever and pharyngalgia followed by a pruritic rash that began on the head and rapidly spread to her trunk and extremities. Clinical examination upon the patient’s admission revealed extensive erythema of the trunk, along with multiple atypical targetoid lesions, some with a bullous centre, distributed across both upper and lower extremities. The palms, soles and facial mucosa were also affected. Nikolsky sign was positive on the back and on the central abdominal region. The patient reported no significant past medical history and had not taken any additional medications during the preceding 3 months. A punch biopsy was performed, which revealed numerous necrotic keratinocytes within the epidermis and subepidermal blistering, confirming the clinical suspicion of SJS/TEN (Figure 1). Over the following days, epidermal detachment progressed to involve >30% of BSA, leading to the final diagnosis of TEN [Common Terminology Criteria for Adverse Events (CTCAE) grade 4, Severity-of-Illness Score for Toxic Epidermal Necrolysis (SCORTEN) 3 (Table 1)]. Treatment was initiated with intravenous methylprednisolone at a dose of 1 mg kg^–1^ daily. Due to the development of new lesions, the dose was escalated to 1.5 mg kg^–1^ daily on the fourth day after the patient’s admission. Disease control was achieved approximately 1 week later, after which a gradual tapering of methylprednisolone was performed over the following 3 weeks. The patient also received topical wound care and supportive treatment in our hospital’s burn unit. Gradual clinical improvement and re-epithelization was observed (Figure 2).

**Figure 1:**
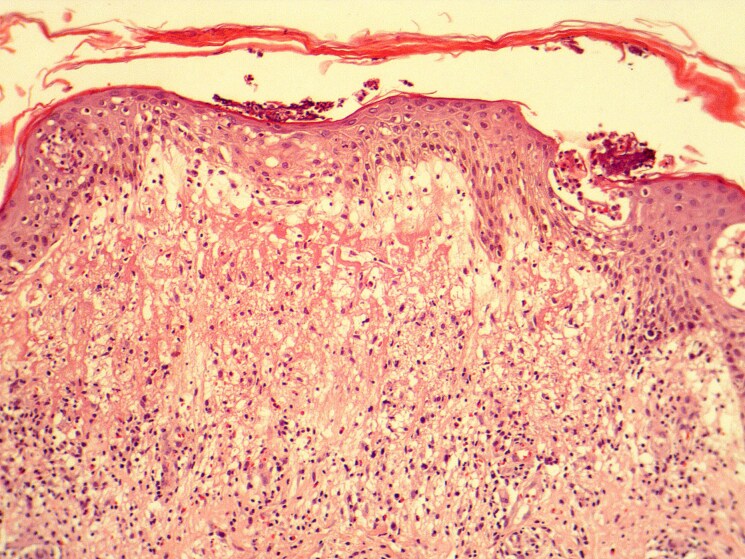
Microscopic image (haematoxylin–eosin, magnification ×40) demonstrating apoptotic keratinocytes within the epidermis, vacuolar degeneration of the basal layer and a lymphocytic infiltrate in the dermis.

**Figure 2:**
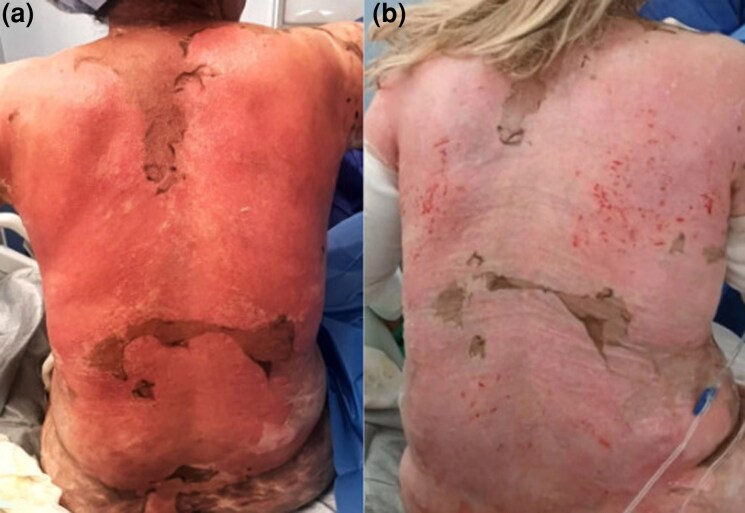
Clinical images showing disease evolution. (a) Diffuse epidermal detachment with underlying erythema at the time of hospital admission; (b) marked clinical improvement with re-epithelialization on day 21 of hospitalization.

The estimated global incidence of SJS/TEN is approximately 1–10 cases per million population per year.^3^ However, large cohort studies have shown that patients with pre-existing malignancies have a higher incidence of these reactions.^3^ Given the rarity of SJS/TEN, robust diagnostic criteria are lacking. Features suggestive of the diagnosis include involvement of at least two mucosal sites, the presence of macular targetoid skin lesions, recent drug exposure and consistent histopathological findings.^2^

Only a few cases in the literature have linked this severe drug reaction to chemotherapy agents. The publications by Huang *et al*. and Then *et al*. describe cases of TEN in patients who received a combination therapy of pemetrexed and cisplatin,^2,3^ whereas Aznab and Khazaei reported TEN in a patient treated with cisplatin in combination with gemcitabine and 5-fluorouracil.^4^ To our knowledge, this is the first reported case of SJS/TEN associated with cisplatin monotherapy.

Cisplatin, an antineoplastic agent that acts through inhibition of DNS synthesis, is the treatment of choice in cervical cancer stages Ib, II III and IVa, either alone or in combination with radiotherapy. Common adverse events include ototoxicity, nephrotoxicity and neurotoxicity.^4^ Radiotherapy is thought to induce keratinocyte damage, making these cells more susceptible to drug-induced reactions. It has also been considered as a potential aggravating factor in the case of TEN, as demonstrated by Aznab and Khazaei.^4^ TEN occurring in patients receiving radiotherapy has usually been linked to anticonvulsant use.^3^

Although the complete immunopathogenesis remains unclear, SJS/TEN is considered a T-cell-mediated type IV (delayed) hypersensitivity reaction.^2^ Immunopathological studies have demonstrated the presence of cytotoxic cells (natural killer T cells and drug-specific CD8^+^ T lymphocytes) within early lesions.^3^ Most cases develop 4–28 days after initial drug exposure,^4^ which is consistent with our case and the previously reported cases, where onset occurred within days to weeks following the first treatment cycle.^2–4^

Cessation of the suspected agent and supportive care, often provided in specialized burn units, seem to be mainstays in treatment of SJS/TEN.^1^ Due to the rarity of the condition, no standardized pharmacological treatment guidelines exist. Given the immunogenic nature of the adverse reaction, several immuno­suppressive therapies have been investigated. High-dose systemic corticosteroids are frequently used. However, the current literature has not demonstrated a statistically significant improvement in patient survival outcomes.^5^ A few studies suggest that intravenous immunoglobulin, either as monotherapy or in combination with systemic corticosteroids, may lead to reduced mortality rates.^1^

Small observational studies have indicated that ciclosporin, when administered to patients with preserved renal function, may also improve mortality outcomes.^1^ Due to their immunosuppressive properties and favourable safety profile, anti-tumour necrosis factor agents have also been trialled, with recent data suggesting that etanercept may shorten the disease course.^5^

Clinicians should be alert to this rare drug reaction, which could be induced by chemotherapy agents such as cisplatin, to ensure prompt drug discontinuation and treatment initiation resulting in improved patient outcomes.

**Table 1:** Severity-of-Illness Score for Toxic Epidermal Necrolysis (SCORTEN) score calculated on the day of the patient’s admission

SCORTEN variables	Patient’s status	Points
Age >40 years	Yes	1
Malignancy	Yes	1
Heart rate >120 bpm	No	0
Initial detachment >10%	No	0
Serum urea >10 mmol L^–1^	Yes	1
Serum glucose >14 mmol L^–1^	No	0
Serum bicarbonate <20 mmol L^–1^	No	0

Total points = 3, estimated mortality risk = 35%. bpm, beats per min.

## Data Availability

The data underlying this article will be shared on reasonable request to the corresponding author.

## References

[1] Frantz R, Huang S, Are A, Motaparthi K. Stevens–Johnson syndrome and toxic epidermal necrolysis: a review of diagnosis and management. Medicina (Kaunas) 2021; 57:895. 34577817 10.3390/medicina57090895 PMC8472007

[2] Huang JJ, Ma SX, Hou X et al Toxic epidermal necrolysis related to AP (pemetrexed plus cisplatin) and gefitinib combination therapy in a patient with metastatic non-small cell lung cancer. Chin J Cancer 2015; 34:94–8. 25418188 10.5732/cjc.014.10151 PMC4360078

[3] Then C, von Einem JC, Müller D et al Toxic epidermal necrolysis after pemetrexed and cisplatin for non-small cell lung cancer in a patient with sharp syndrome. Onkologie 2012; 35:783–6. 23207626 10.1159/000345109

[4] Aznab M, Khazaei M. Stevens-Johnson syndrome patient received combination chemotherapy gemcitabine, cisplatin, and 5-FU for biliary tract cancer. Iran J Cancer Prev 2016; 9:e4211. 27703643 10.17795/ijcp-4211 PMC5038831

[5] Canhão G, Pinheiro S, Cabral L. Toxic epidermal necrolysis: a clinical and therapeutic review. Eur Burn J 2022; 3:407–24. 39599955 10.3390/ebj3030036 PMC11571860

